# Physicians’ beliefs about placebo and nocebo effects in antidepressants – an online survey among German practitioners

**DOI:** 10.1371/journal.pone.0178719

**Published:** 2017-05-31

**Authors:** Lea Kampermann, Yvonne Nestoriuc, Meike C. Shedden-Mora

**Affiliations:** 1 Department of Systems Neuroscience, University Medical Center Hamburg-Eppendorf, Hamburg, Germany; 2 Department of Psychosomatic Medicine and Psychotherapy, University Medical Center Hamburg-Eppendorf, Hamburg, Germany; Tokai University, JAPAN

## Abstract

**Background:**

While substantial placebo and nocebo effects have been documented in antidepressant clinical trials, physicians’ awareness of the nonspecific effects in routine antidepressant treatment remains unclear. The study investigated physicians’ beliefs and explanatory models regarding the desired effects and undesired side effects of antidepressants, with specific emphasis on nonspecific effects accounted for by placebo and nocebo mechanisms.

**Methods:**

An online survey was conducted among 87 physicians (40.2% psychiatrists, 25.3% neurologists, 24.1% general practitioners, 12.6% internists, 21.8% other). The survey assessed the physician’s beliefs in antidepressant effectiveness, as well as 6 explanatory models regarding antidepressant effectiveness and 8 explanatory models for the occurrence of side effects.

**Results:**

Most physicians (89.7%) believed in the effectiveness of antidepressants while acknowledging a considerable role of the placebo effect by attributing around 40% of the total effects to nonspecific factors. For both antidepressant effectiveness and the occurrence of side effects, pharmacological effects were rated as most important (93.1% and 80.5% agreement), but physicians also attributed a substantial role to the patients’ expectations (63.2% and 58.6%) and experiences (60.9% and 56.3%). Concerning the physician’s own role in promoting nonspecific effects in antidepressant effectiveness, highest endorsements were found for the quality of the physician-patient-relationship (58.6%) and own expectations (41.4%). When asked about side effects, fewer participants agreed that informing the patient about known side effects (25.2%) or the physicians’ expectations themselves (17.2%) could induce side effects.

**Conclusion:**

Physicians, when prescribing antidepressants, are generally open towards nonspecific treatment mechanisms. However, they consider their own influence as less important than the patient’s side, especially when it comes to the explanation of unwanted side effects. Awareness of the possible beneficial as well as malicious role of nonspecific mechanisms should be fostered as the first step towards optimizing antidepressant treatment by promoting placebo while avoiding nocebo effects.

## Introduction

In the last decade, prescription rates of antidepressants have constantly and strongly increased [1]. The main reason to prescribe antidepressants is a given indication defined by official guidelines, as it would be the case for a manifest severe depression. However, limitation of other treatment resources or meeting a patient’s wish have also been named as prescription reasons [2]. In an online survey, the majority of physicians (76%) reported that they had at least once prescribed different forms of treatments “in situations without demonstrated or expected clinical efficacy” [3]. Moreover, 38% of the psychiatrists reported the prescription of subtherapeutic doses of antidepressants, indicating “a dosage less than the amount required for a therapeutic effect” (Subtherapeutic, 2011 as cited in [4]). By prescribing a dosage that is not expected to have a therapeutic effect, an underlying intention to aim for non-pharmacological mechanisms of effectiveness appears self-evident. Antidepressants are thus not always prescribed for pharmacological reasons only.

A drug’s overall effect is considered to result from two main mechanisms of effectiveness: the pharmacological, i.e. bio-physiological effects caused by a drug’s chemical ingredients on the one hand, and another, nonspecific mechanism on the other hand. The latter presumes effects resulting from experience, expectancy as well as context factors, such as the patient-physician-interaction. Whenever nonspecific effects appear as beneficial outcome, such as symptom reduction, they are referred to as placebo effects [5], while for nonspecific effects causing unwanted side effects, the corresponding term of nocebo has been established [6].

In antidepressants, these placebo effects account for up to 75% of antidepressants’ total effectiveness [7,8] and vary considerably according to study design, publication year or assessment method [9,10]. Thus, placebo effects represent a potent mechanism in antidepressants’ effectiveness and should be appropriately considered when prescribing these. However it remains unclear to which degree the concept of benefits resulting from placebo effects has reached the medical practice insofar that physicians harness these effects within the prescription of antidepressants. Until today, corresponding research beyond classical clinical trials involving placebo arms remains scarce.

Up to now, most of these studies addressing the utilization of placebo effects in the daily practice only refer to the use of actual placebos, i.e. pills without any active pharmaceutical ingredient. Corresponding findings on physicians’ attitudes report a general belief that placebos have a therapeutic effect as well as the willingness to utilize them where research has proven their efficacy [11]. Further studies found patients to be open towards the use of placebos when administered in an appropriate, i.e. transparent manner; yet, this disposition is underestimated by most physicians [7,8]. Even for depression, patients have been found to be open towards placebo treatment without considering it to be a deceit [12]. Overall, the available studies show that patients as well as physicians are aware of the placebo effect and consider it a possible factor within the spectrum of medical treatments, even though clear guidelines as well as practical experience are still missing in daily practice. However, studies going beyond the administration of placebos, but investigating placebo components (i.e. contexts effects) during application of active drugs remain rare. In a recent study [13], Vijapura and colleagues found that psychiatrists consider on average 26% of antidepressants’ clinical outcomes as due to placebo effects, thus underestimating the assumed empirical evidence for nonspecific effects in antidepressants nearly threefold [14,15]. Besides, Vijapura et al. found that the contribution of pharmacological ingredients in antidepressant effects was rated higher than patient and clinician characteristics [13]. The strong favor for pharmacological explanations of effects was however reduced in physicians that graduated from medical school more recently, pointing at the novelty of these considerations in pharmacotherapy.

Parallel to explanatory models for a drug’s effectiveness, it is assumed that the occurrence of side effects is determined by nonpharmacological effects as well [16]. As in the placebo effect, the patient’s expectancy shaped by treatment information, social influences and prior experience might actually cause the occurrence of unwanted side effects [17]. This nocebo effect can not only cause adverse effects but might even impair a drug’s therapeutic efficacy by decreasing the patient’s adherence to take the medication as recommended [6,18,19]. In antidepressants, substantial nocebo effects with strong impact on adherence, treatment and treatment efficacy have been found [20,21]. Thus, health care professionals should be aware of these mechanisms and avoid them whenever possible. Still, to our knowledge, there are no studies so far investigating physicians’ or patients’ awareness of these effects in antidepressants.

With a growing body of evidence for the clinical relevance of nonspecific mechanisms in a drug’s efficacy, leading researchers call to enhance placebo effects and minimize nocebo effects to improve health care [5,16,22,23]. Also in antidepressants, efficacy could be maximized by intentionally embracing nonspecific mechanisms as expectation, experience and context factors in the drug’s administration, whereas negative side effects should be reduced by avoiding the same mechanisms causing unwanted effects.

Concerning the use of antidepressants, it is not yet widely studied to which degree physicians consider placebo and nocebo effects in their practical work. Do they estimate the nonspecific (i.e. placebo) effect as high as clinical studies do? And furthermore, are they aware of their own possible contributions, such as the effect of their relationship to the patient, wording or other possible suggestive influences? While Vijapura et al. [13] present first evidence on the behalf of placebo effects being considered in the use of antidepressants, beliefs about nocebo responses as explanatory factor for the occurrence of side effects remain unstudied. Therefore, besides placebo components, this study also investigates whether physicians are aware of the nocebo effect inducing side effects by nonspecific mechanisms such as expectancy and experience. Being aware of the potent impact that nonspecific effects might have both on the drug’s efficacy as well as the development of side effects is a necessary precondition to enable positive and avoid negative effects evolving from these factors. Finding low awareness of these factors in doctors representing daily practice would then call for more educational work, enabling doctors to maximize placebo and minimize nocebo effects whenever possible, thus substantially improving health care.

The present study aimed to explore physicians’ beliefs and explanatory models about possibly related nonspecific placebo and nocebo effects in antidepressant effectiveness and the occurrence of side effects. Moreover, the relative importance attributed to pharmacological vs. nonspecific mechanisms, and patient vs. physician factors was analyzed.

## Methods

### Online survey

The presented data was collected in an online survey using Unipark Software. Data was collected from November 2013 to January 2014. Primary care physicians, psychiatrists, neurologists and internists working in hospitals or private practices and regularly prescribing antidepressants were invited to participate via e-mails and print letters, which were sent to (e-mail) addresses found on the websites of medical associations, as a membership herein is obligatory in Germany. Printed letters were sent only to practices that did not list an email address and were residential in the Hamburg metropolitan area. Approximately 800 physicians were invited to participate (600 physicians were contacted via e-mail, 200 print letters sent out). As the recruitment process was rather open, i.e. the link could be shared among colleagues, it is not possible to determine the exact number of physicians invited to participate. The possibility of a selection bias is addressed in the limitations section.

The questionnaire asked for participants’ socio-demographic information such as gender, age, year of obtaining their MD, work experience in years, employment setting (hospital or private practice), field of profession, position, diploma of specialization and whether they mainly treated adults or children.

Physicians’ attitudes towards effectiveness were assessed using a single question (‘How effective do you consider antidepressants in general?’) answered on a likert-scale ranging from 1 (‘ineffective’) to 5 (‘effective’). Subsequently, two rating sliders ranging from 0 to 100% were used to quantify how much of antidepressants’ total effectiveness is attributed to their pharmacological profile or the placebo effect, respectively.

Physicians’ explanatory models towards six different mechanisms of antidepressant effectiveness were assessed on a likert-scale ranging from 1 (‘fully disagree’) to 5 (‘fully agree’). They were asked how much they agreed that the following mechanisms played a role in the effectiveness of antidepressants: the pharmacological effect, the patient’s expectations, the patient’s former experience, and the physician’s expectations, the physician-patient-relationship and spontaneous recovery over time.

Subsequently, physicians’ explanatory models for the occurrence of side effects were assessed on a likert-scale from 1 (‘never’) to 5 (‘always’). Supposed explanatory models were: the pharmacological profile, the patient’s expectations, the patient’s former experiences, the physician’s expectations and the physician inducing them by informing about known side effects when prescribing AD, e.g. during an informed consent procedure.

Since practitioners' beliefs on mechanisms of antidepressant efficacy are a novel field of inquiry, the present questionnaire was designed for exploratory use in the current study. It has not been validated in an independent sample.

Approval of the Ethics Commission of the German Psychological Society (DGPs) was obtained prior to starting the study. All participants gave informed consent by accepting an informed consent statement shown previous to providing any research responses. Participants did not receive any financial incentive. Completion of the online survey took approximately 5 minutes.

### Statistical analyses

Rating sliders on attributed proportions of pharmacological and nonspecific effects in AD were not restricted to sum up to 100%, in order to allow for additional explanatory factors that physicians might have in mind contributing to the overall effectiveness. This resulted in the sum of some subjects summing up to more than 100%. Besides descriptive characteristics, the percentage of physicians who at least partly agreed was calculated for each statement. In order to contrast the relative importance of pharmacological vs. nonspecific mechanisms, and patient vs. physician factors, Bonferroni corrected paired t-tests were conducted based on means, and effect sizes were reported.

## Results

### Sample

Out of 146 physicians who initiated the online questionnaire, eighty-seven physicians (59.6%) completed it. They were between 23 and 70 years old (45.8 ± 10.7 mean (M) ± standard deviation (SD)), 49.4% of them female, 75% working in a city. The majority (82%) had completed a medical specialty training, of which 40.2% were psychiatrists, 25.3% neurologists, 24.1% general practitioners and 12.6% internists respectively. 50.5% of the sample were working in an ambulatory primary care setting, 47.1% in an in-patient clinic. Mean working experience was 17.5 ± 10.3 years (M ± SD).

### Physicians’ explanatory models for antidepressant effectiveness

Concerning the effectiveness of antidepressants, 89.7% of the physicians considered antidepressants to be effective, 9.2% were undecided and one participant (1.1%) rated them as rather ineffective. Out of the total effectiveness, 59.8% (95% confidence interval (CI) = [56.1, 63.4] were attributed to antidepressants’ pharmacological profile using the rating slider, whereas the placebo effect contributed 41.5% (95% CI = [37.6, 45.5] (The sum of both sliders exceeded 100% in some cases).

**Fig 1:**
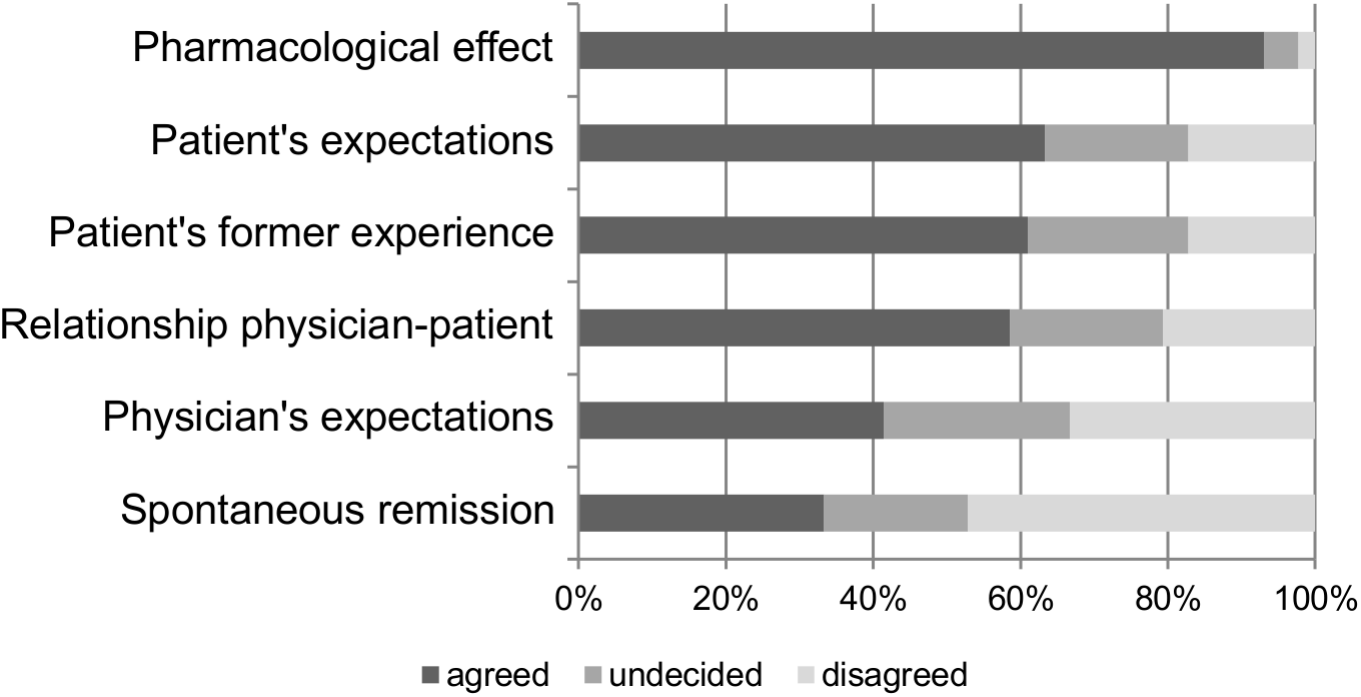
Physicians’ agreement to different explanatory models for antidepressant effectiveness. Depicted is the percentage of participants who agreed, disagreed or were undecided whether they agreed to the suggested mechanisms of effectiveness for antidepressants.

Regarding explanatory models, Fig 1 shows the percentage of physicians who agreed to the five suggested mechanisms of antidepressants’ effectiveness. As can be seen, pharmacological effects were rated as most important and showed the highest rate of agreement (93.1%). Of the total sample, 63.2% rated the patient’s expectations as important, as well as the patient’s former experience (60.9%). Furthermore the relationship between physician and patient was also rated as accepted mechanism of effectiveness, as 58.6% of all physicians agreed. Moreover, 41.4% of the physicians recognized their own expectations as a possible mechanism of effectiveness. Only one third (33.3%) of the participants also considered the depression’s spontaneous remission as part of an antidepressant’s effectiveness.

Contrasting the relative importance of different suggested mechanisms, the pharmacological profile was given the highest importance as mechanism of effectiveness, as compared to all other nonspecific mechanisms (t = 5.30–8.27, all p < .001, d = 0.77–1.32), as well as the spontaneous remission (t = 9.12, p < .001, d = 1.50). When comparing the perceived contribution of patient factors to physician factors, the patient’s expectations were rated as contributing more than the physician’s expectations (t = 5.26, p < .001, d = 0.52), also the patient’s former experience was considered as more relevant when compared to the physician’s expectations (t = 4.78, p < .001, d = .50). The other comparisons did not differ significantly.

Summarizing, physicians’ endorsement of the pharmacological explanatory model succeeded nonspecific factors substantially, as the vast majority of physicians agreed to the pharmacological effect in antidepressants and attributed more than half of overall effectiveness to it. However, more than half of physicians also endorsed nonspecific factors such as experience and expectation, as well as physician-patient relationship as explanatory model for AD effectiveness, while own contributions were only stated relevant by less than half of the sample, and were even rejected by one third.

### Physicians’ explanatory models for the occurrence of antidepressant side effects

**Fig 2:**
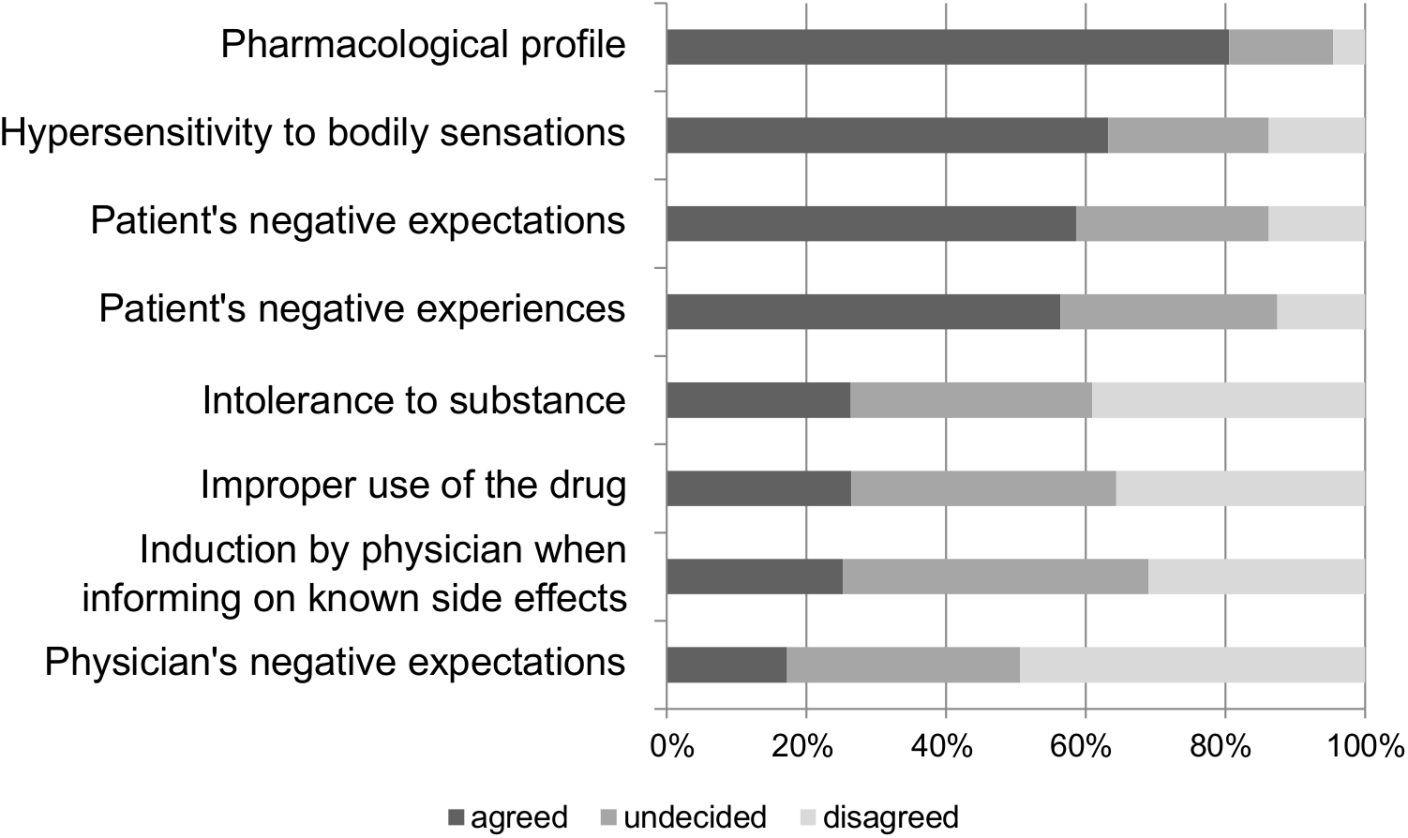
Physicians’ agreement to different explanatory models for the occurrence of side effects from antidepressants. Depicted is the percentage of participants that agreed, disagreed or were undecided whether to endorse the suggested reasons for the occurrence of side effects in antidepressants.

A similar pattern was found in the analysis of physicians’ explanatory models for side effects (Fig 2). Pharmacological causes met the highest approval rate of 80.5%. Hypersensitivity to bodily sensations, as well as patient’s expectations and experiences showed medium to high levels of approval (63.2%, 58.6% and 56.3%). Physicians agreed to a lower extent to substance intolerance and improper use of the drug (both 26.4%) as causes for side effects. The lowest approval was found in explanatory factors regarding the physician’s own contribution, i.e. inducing side effects by talking about known side effects when describing AD (25.3%), as well as the physician’s own expectation of side effects (17.2%).

Comparing the relative importance of explanatory factors for side effects, the pharmacological profile was rated as most important, when compared to all other factors (t = 2.20–10.41, p = 0.03 - < 0.001, d = 0.29–1.40). All patient factors, except for substance intolerance as well as improper use of the drug, were rated as more relevant than factors that implied the physician’s involvement, such as the informing about known side effects (t = 5.16–6.36, p < .001, d = 0.58–0.69) or physicians`expectations (t = 8.67–9.53, p < .001, d = 0.89–1.00). The physicians’ own expectations were furthermore rated less relevant than induction by informing about known side effects (t = 2.98, p = .004, d = 0.32).

Summarizing, we found that the pharmacological profile was endorsed by the vast majority of physicians, and patient factors such as expectation and experience were rated relevant by over the half of the sample, with only a small number of actual rejections. Factors regarding the physicians’ own impact, such as informing on known side effects as well as the physicians’ own expectations were rejected by more physicians than endorsed.

Results illustrated separately for different medical specialties (psychiatrists, neurologists, general practitioners, internists) are available in the supplementary information (S1 Fig).

## Discussion

The presented study assessed physicians’ explanatory models for beneficial effects and side effects of antidepressants. Ninety percent of the participants endorsed the general effectiveness of antidepressants. Thus, this study documents physicians’ general belief in the benefits of antidepressant treatment. The physicians’ explanatory models for antidepressant effectiveness showed clear favors towards a pharmacological factor. Beyond this, the majority of the participating physicians were aware of the importance of placebo effects in antidepressant effectiveness. They stated their belief in psychological factors such as the patient’s expectations and prior experience, as well as the patient-physician-relationship. However, they did not equally acknowledge the importance of physician’s own expectations.

Pharmacological factors were also the most prominent explanation for side effects of antidepressants. Psychological factors on the patient side, such as expectation, experience and hypersensitivity to bodily changes also showed a high rate of approval. However, physicians rather disapproved of items suggesting the physician’s possible contribution to side effects, such as their own expectations (49.4% denied) and informing about known side effects (31% denied).

Overall, the findings suggest that the majority of physicians is generally aware of placebo and nocebo effects in antidepressants caused by psychological factors such as expectation and experience from patient and–to a lower degree–physician. However, these effects are given a subsidiary role when compared to pure pharmacological effects. Specifically, their contribution is rated lower than evidence-based data synthesis from clinical studies with placebo arms suggest (39% in our study vs. 68–75% in meta-analyses [15,24]). Although these meta-analyses are based on placebo administration instead of placebo effects within the application of active drugs, we consider it a helpful benchmark to frame the estimation of nonspecific mechanisms of efficacy in our sample. This discrepancy might suggest that physicians underestimate possible placebo effects in antidepressants, which is in line with Vijapura et al.’s conclusion [13], even though in our data this is less pronounced. Furthermore, our study shows that the physician’s own contribution is rated more important when considering positive outcomes as compared to side effects.

The finding that at least a proportion of physicians is aware of their own impact on drug effectiveness as well as potential side effects is both novel and intriguing, as physicians’ beliefs are rarely subject of medical research, despite of their significant impact on drug efficacy. Doctors’ awareness of nonspecific effects might be a first and necessary step towards harnessing these mechanisms for the benefit of the patient, as it might induce stronger positive responses to the medication. This is underlined by the publication year effect showing stronger placebo effects in more recent studies than in earlier research, where awareness of placebo effects had not been established to a comparable amount [9,10]. The more physicians know about possible placebo effects and their role in inducing them, the more patients might be able to benefit. There is increasing evidence showing that physicians who are aware of context effects are intentionally as well as unintentionally able to increase placebo effects of a given drug, given that being more convinced themselves might render them more convincing for others [5,22,25]. The fact that many of the participants stated their general belief in nonspecific drug factors supports the notion that placebo and nocebo effects are not considered as something opposed to “true” pharmacological effects, but instead established as a potent explanatory factor. The awareness of these factors within daily practice, i.e. going beyond scientific research, is favorable as it allows promoting effects to the patients’ benefit. However, as not all of the physicians endorsed these factors and many were undecided, there is considerable room for improvement, which could and should be captured in medical teaching and training.

Furthermore, many of the participants disagreed with the thought of inducing side effects by informing about known side effects in AD, or by own expectations. As the nocebo effect is a rather recent research field [19], it might explain the awareness of the own contribution remaining less pronounced as compared to placebo. However, it is important to underline the consequences of physicians underestimating their own impact on side effects: not only might it induce side effects by inconsiderately transferring information on known side effects to patients when prescribing AD, but also reduce the patient’s compliance, and thus holds clinical implications [18,26].

At this point it remains an open question whether physicians make intentional use of placebo effects when prescribing antidepressants, pointing at a “placebo intention”: Do physicians go so far as to prescribe antidepressants out of a placebo intention, while not explicitly indicated? Or does the belief in placebo effects in antidepressants simply lead physicians to enhance nonspecific placebo effects and to reduce the impact of nocebo-related medication side effects? These questions should be answered in further studies investigating physicians’ intentions in addition to explanatory models when prescribing antidepressants.

As a possible limitation to the generalizability of the results, a selection bias has to be considered inherent in the way that physicians’ contacts were accessed (see method section), withholding us from determining the exact number of physicians invited to participate As physicians participated in this online survey without any financial compensation, there might be a self-selection towards physicians with high interest in the topic and/or affinity to use the internet. Furthermore, presenting a plurality of possible explanatory factors might have been more suggestive than a free recall of personal beliefs. However, the high rate of disapproval for controversial items, such as inducing side effects by informing the patient about them, shows that participants did not generally overstate their agreement.

## Conclusion and future directions

In order to benefit most from nonspecific mechanisms of effectiveness, it is important to establish a culture in the daily medical practice, in which physicians know of and believe in the potential of these mechanisms, and consider the patient’s possible openness towards them [7,8]. The same thought is valid for considering and avoiding nocebo effects, which would provide a useful possibility to reduce side effects [16]. We believe that the targeted utilization of nonspecific effects has the potential to improve antidepressant effectiveness for guideline-defined indications at low costs [27].

Possible approaches could include embracing beneficial placebo effects by modulating the patient’s expectations. This could be achieved by verbal suggestions regarding the proven effectiveness of the given medication [28–30], by shaping positive expectations to the treatment’s outcomes [31–33] or by disputing the patient’s beliefs and experiences with former medication. Furthermore, physicians should be well aware of the importance of the patient-physician relationship [34], thus aiming to create a beneficial, empathetic environment when in contact with patients. Further studies and educational work is needed to guide physicians towards a profound understanding of these mechanisms and their exploitation for the patients’ benefit.

## Supporting information

S1 Appendix Survey questionnaire (English version). (DOCX)

S2 Appendix Survey questionnaire in original language (German version). (DOCX)

S1 Fig Contrasting results for different medical specialties. Color coding was chosen to differentiate between specialties of participants, where red = psychiatry (n = 35), green = neurology (n = 22), blue = general medicine (n = 21) and brown = internists (n = 11), whereas these colors were shaded to code agreement (darker) vs. disagreement (brighter), just as introduced in Figs 1 and 2 of the result section. (A) Ratings on general effectiveness of antidepressants (AD) (agreed, undecided, disagreed) (B) Estimated proportion of pharmacological (pharm) vs. placebo (plac) responses in ADs' overall effectiveness ([%], M ± SEM). (C) Endorsement of six different explanatory factors for ADs' effectiveness. (D) Rated relevance of eight explanatory factors for the development of side effects in AD.
(TIF)
