# Supplementary material for: Physicians’ beliefs about placebo and nocebo effects in antidepressants – an online survey among German practitioners
Source: PLoS One. 2017 May 31;12(5):e0178719. doi: 10.1371/journal.pone.0178719 (PMC5451122; doi:10.1371/journal.pone.0178719)
Supplement: S1 Appendix — (DOCX) [file pone.0178719.s001.docx]

# General Questions

## Gender

- female
- male

## Age

## Year of approbation

## Years of professional medical activity

## Please specify your form of employment

- hospital employment
- own practice
- employed in a practice
- other _________________

## Are you working as a general practitioner?

- yes
- no

## Please indicate your specialty (multiple selections possible)

- psychiatry
- neurology
- general medicine
- internal medicine
- other _________________

## Please indicate your job position

- intern/resident
- attending
- chief
- none of these options applies.
- other _________________

## Do you have a completed specialty training?

- yes
- no

# Survey on antidepressants

|  | ineffective | partly ineffective | undecided | partly effective | effective |
| --- | --- | --- | --- | --- | --- |
| How effective do you consider antidepressants to be in general? | 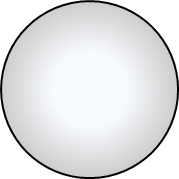 | 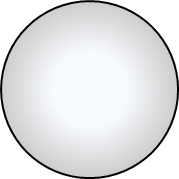 | 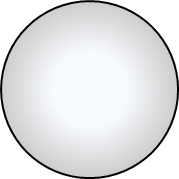 | 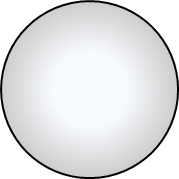 | 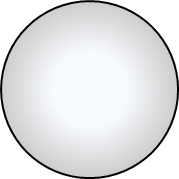 |

## How much do you agree with the following statements to the effectiveness of antidepressants?

| **Antidepressants are effective** … | fully disagree | partly disagree | undecided | partly agree | fully agree |
| --- | --- | --- | --- | --- | --- |
| … because of their pharmacological profile | 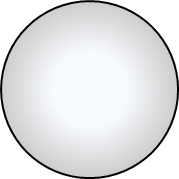 | 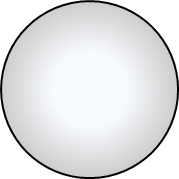 | 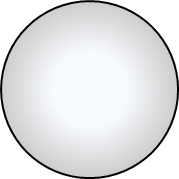 | 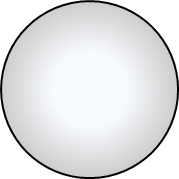 | 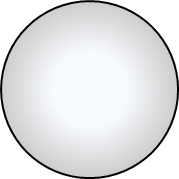 |
| … because the patient expects their effectiveness. | 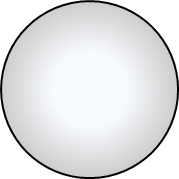 | 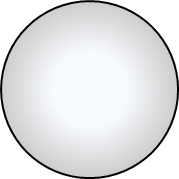 | 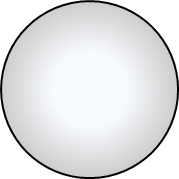 | 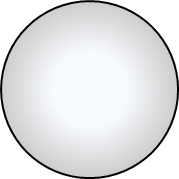 | 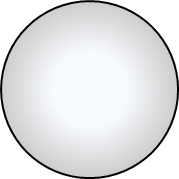 |
| .. because the patient has made the experience that drugs lead to a symptom reduction. | 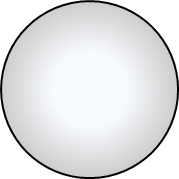 | 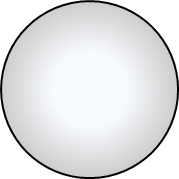 | 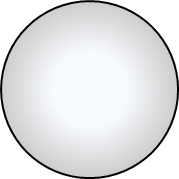 | 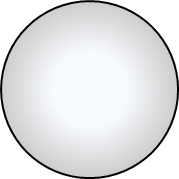 | 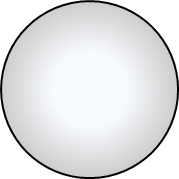 |
| … because the treating practitioner expects the drug to be effective. | 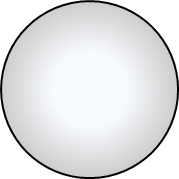 | 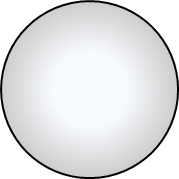 | 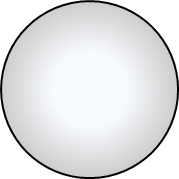 | 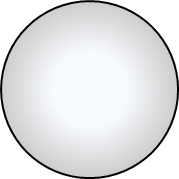 | 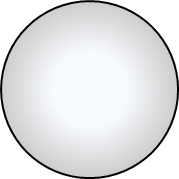 |
| … because the relationship between physician and patient acts beneficially on the patient's well-being. | 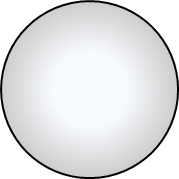 | 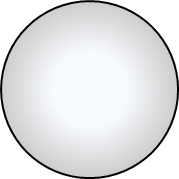 | 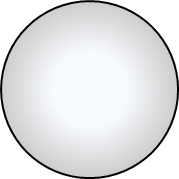 | 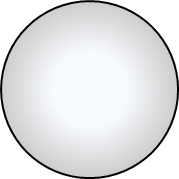 | 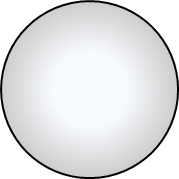 |
| … because depression improves over time, with or without antidepressants. | 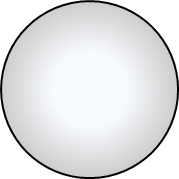 | 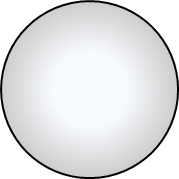 | 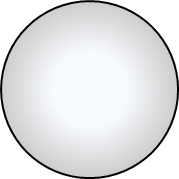 | 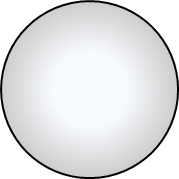 | 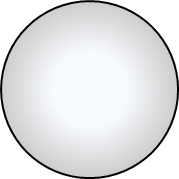 |

## Within all medical treatments, both pharmacological mechanisms (e.g. Serotonine-Reuptake-Inhibition) as well as nonspecific (placebo effects, e.g. positive expectations) of mechanisms efficacy play a role.

How high do you estimate the proportion of overall antidepressants' effectiveness that is induced by pharmacological mechanisms?


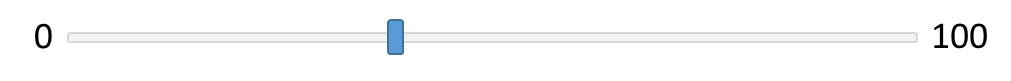


How high do you estimate the proportion of overall antidepressants' effectiveness that is induced by nonspecific mechanisms (placebo effects)?


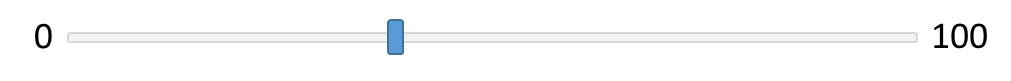


**Some patients develop unwanted side effects during the administration of antidepressants. Which reasons do you consider to be responsible for these side effects?**

Please indicate how often you think the following reasons are relevant factors for side effects.

|  | never | rarely | sometimes | often | always |
| --- | --- | --- | --- | --- | --- |
| Improper use of the drug | 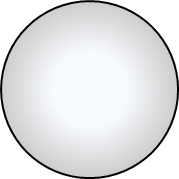 | 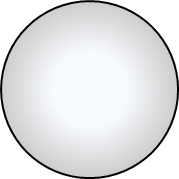 | 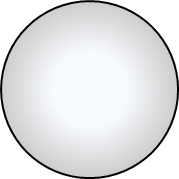 | 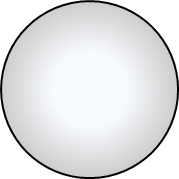 | 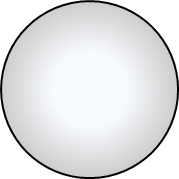 |
| Pharmacological profile of the drug | 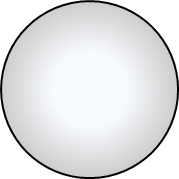 | 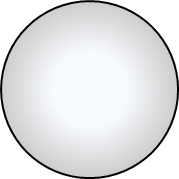 | 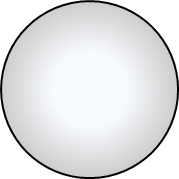 | 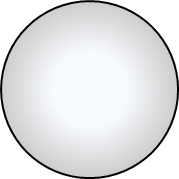 | 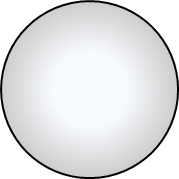 |
| Patient's negative experiences | 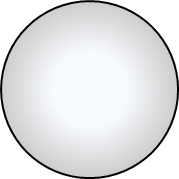 | 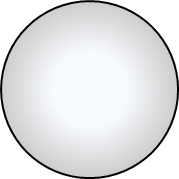 | 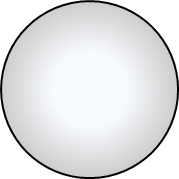 | 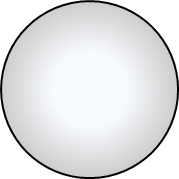 | 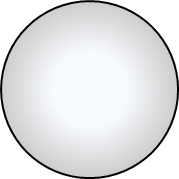 |
| Patient's negative expectations | 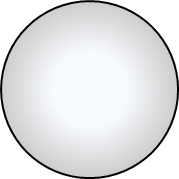 | 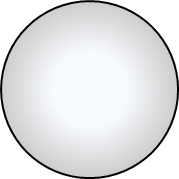 | 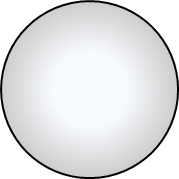 | 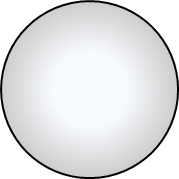 | 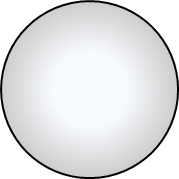 |
| Physician's negative expectations | 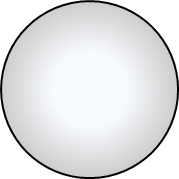 | 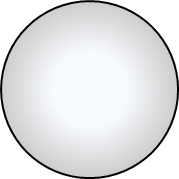 | 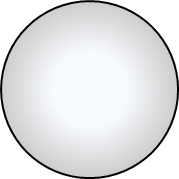 | 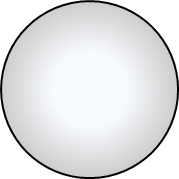 | 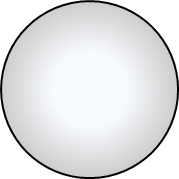 |
| Induced by physician informing patients on known side effects when prescribing AD | 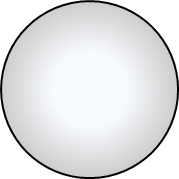 | 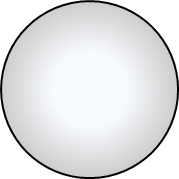 | 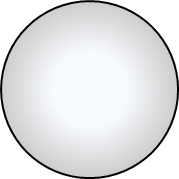 | 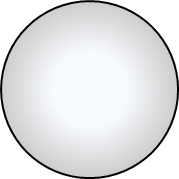 | 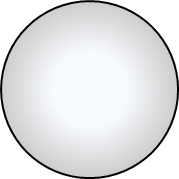 |
| Hypersensitivity to bodily sensations in these patient groups | 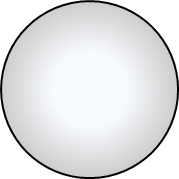 | 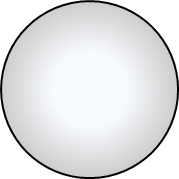 | 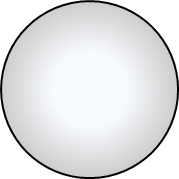 | 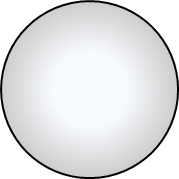 | 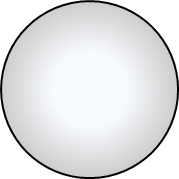 |
| Intolerance to certain substances | 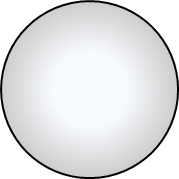 | 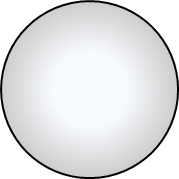 | 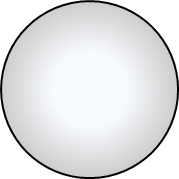 | 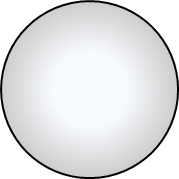 | 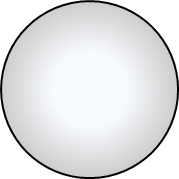 |
