# Supplementary material for: Physicians’ beliefs about placebo and nocebo effects in antidepressants – an online survey among German practitioners
Source: PLoS One. 2017 May 31;12(5):e0178719. doi: 10.1371/journal.pone.0178719 (PMC5451122; doi:10.1371/journal.pone.0178719)
Supplement: S2 Appendix — (DOCX) [file pone.0178719.s002.docx]

# Allgemeine Fragen

## Geschlecht

- weiblich
- männlich

## Alter

## Jahr der Approbation

## Ärztliche Tätigkeit in Jahren

## Bitte geben Sie dir Art Ihrer Beschäftigung an

- in Krankenhaus angestellt
- niedergelassen in Praxis
- in Praxis angestellt
- Sonstiges: _________________

## Sind Sie in der hausärztlichen Versorgung tätig?

- ja
- nein

## Bitte geben Sie Ihre Fachrichtung an (Mehrfachantworten möglich)

- Psychiatrie
- Neurologie
- Allgemeinmedizin
- Innere Medizin
- Sonstiges: _________________

## Bitte geben Sie Ihren Berufsstatus an

- Assistenzarzt
- Oberarzt
- Chefarzt
- Nicht davon trifft zu.
- Sonstiges: _________________

## Haben Sie eine abgeschlossene Facharztausbildung?

- ja
- nein

# Befragung zu Antidepressiva

|  | unwirksam | eher unwirksam | unentschieden | eher wirksam | wirksam |
| --- | --- | --- | --- | --- | --- |
| Für wie wirksam halten Sie Antidepressiva im Allgemeinen? | 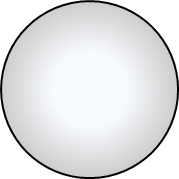 | 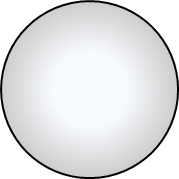 | 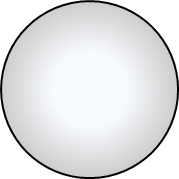 | 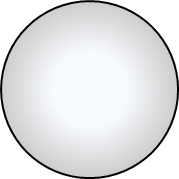 | 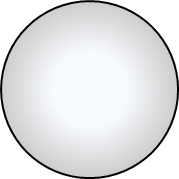 |

**Inwieweit stimmen Sie den folgenden Aussagen zur Wirkung von Antidepressiva zu?**

| **Antidepressiva wirken …** | stimme nicht zu | stimme eher nicht zu | unentschieden | stimme eher zu | stimme zu |
| --- | --- | --- | --- | --- | --- |
| … durch den pharmakologischen Effekt. | 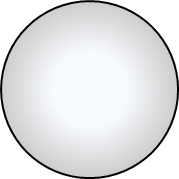 | 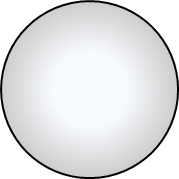 | 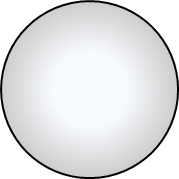 | 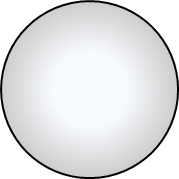 | 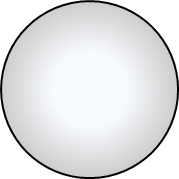 |
| … da der Patient die Wirkung des Präparates erwartet. | 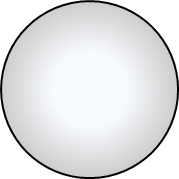 | 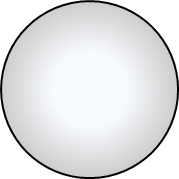 | 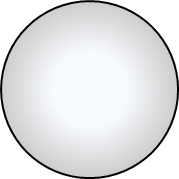 | 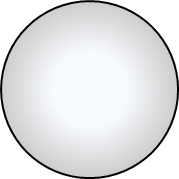 | 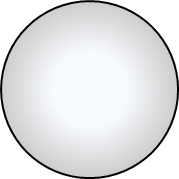 |
| …, weil der Patient in der Vergangenheit die Erfahrung gemacht hat, dass Medikamente zu einer Verbesserung seiner Symptome führen. | 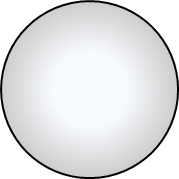 | 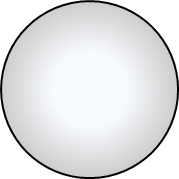 | 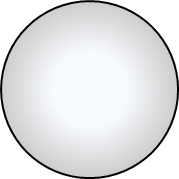 | 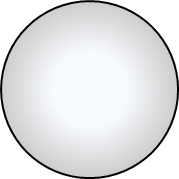 | 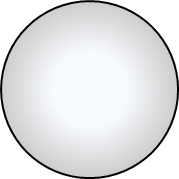 |
| …, weil der verordnende Arzt erwartet, dass das Präparat wirken wird. | 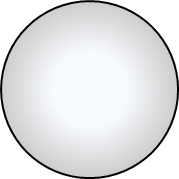 | 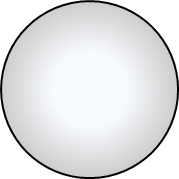 | 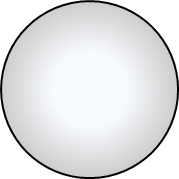 | 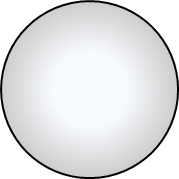 | 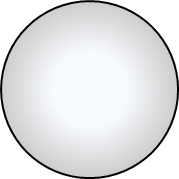 |
| …, weil der Kontakt zwischen Arzt und Patient sich positiv auf das Befinden des Patienten auswirkt. | 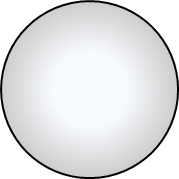 | 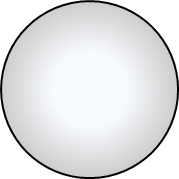 | 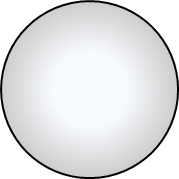 | 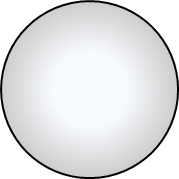 | 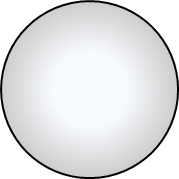 |
| … da Depressionen mit der Zeit besser werden – ob mit oder ohne Antidepressiva. | 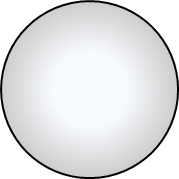 | 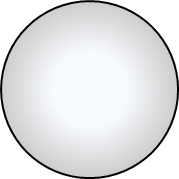 | 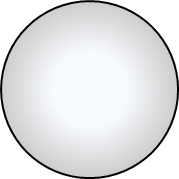 | 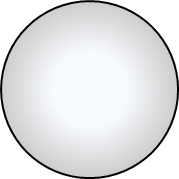 | 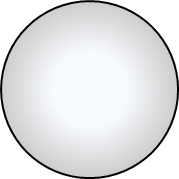 |

**Bei allen medizinischen Behandlungen spielen sowohl pharmakologische Wirkmechanismen (z.B. Wiederaufnahmehemmung von Serotonin) als auch unspezifische (Placebo-Effekte, z.B. positive Erwartung) eine Rolle.**

Wie hoch schätzen Sie den Anteil der Wirkung bei Antidepressiva ein, der durch den pharmakologischen Wirkmechanismus hervorgerufen wird?


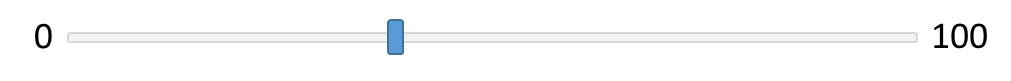


Wie hoch schätzen Sie den Anteil des unspezifischen Wirkmechanismus (Placebo-Effekt) bei der Wirkung von Antidepressiva ein?


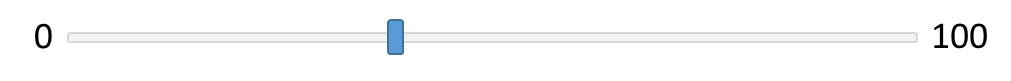


**Einige Patienten entwickeln bei der Antidepressiva-Einnahme Nebenwirkungen. Welche Gründe gibt es Ihrer Meinung nach für die Entwicklung von Nebenwirkungen?**

Bitte geben Sie an, wie oft die genannten Gründe Ihrer Ansicht nach eine Rolle spielen.

|  | nie | selten | manchmal | oft | immer |
| --- | --- | --- | --- | --- | --- |
| Nicht ordnungsgemäße Einnahme | 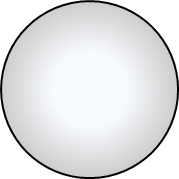 | 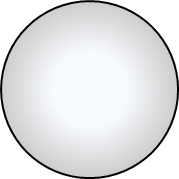 | 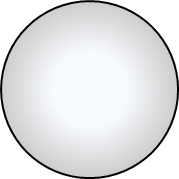 | 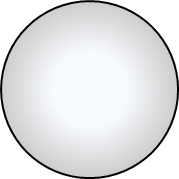 | 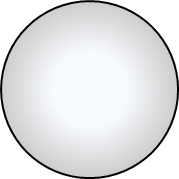 |
| Pharmakologisches Wirkprofil des Medikaments | 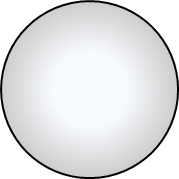 | 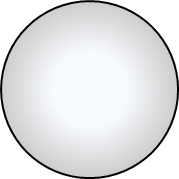 | 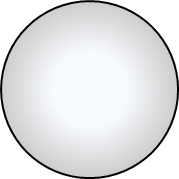 | 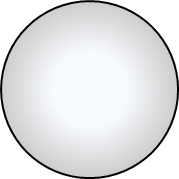 | 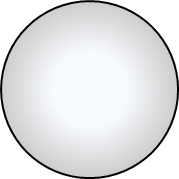 |
| Negative Erfahrungen des Patienten | 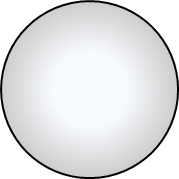 | 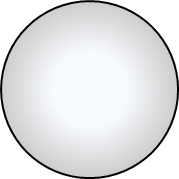 | 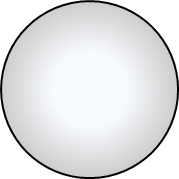 | 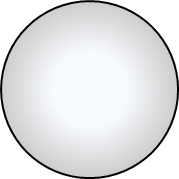 | 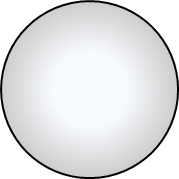 |
| Negative Erwartungen auf Seiten des Patienten | 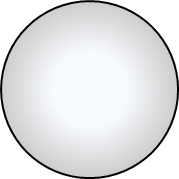 | 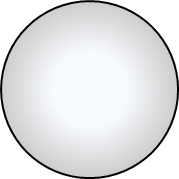 | 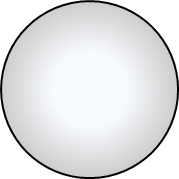 | 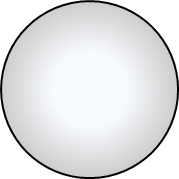 | 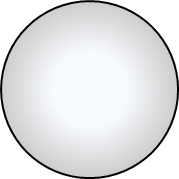 |
| Negative Erwartungen auf Seiten des Arztes | 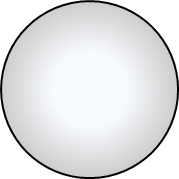 | 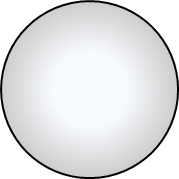 | 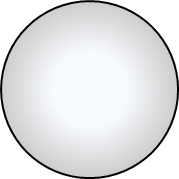 | 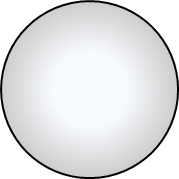 | 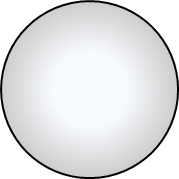 |
| Durch die Aufklärung des Arztes induziert | 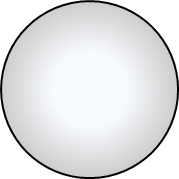 | 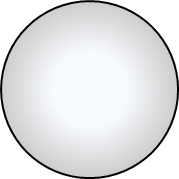 | 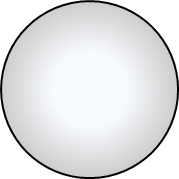 | 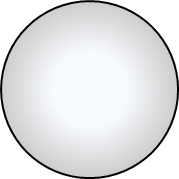 | 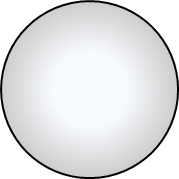 |
| Verstärkte Sensitivität für Körperempfindungen bei diesen speziellen Patientengruppen | 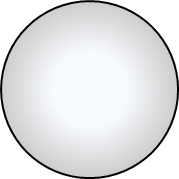 | 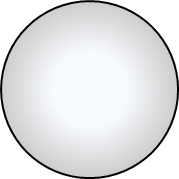 | 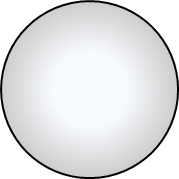 | 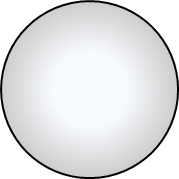 | 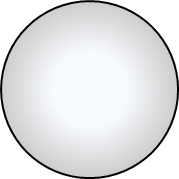 |
| Unverträglichkeit gegen bestimmte Inhaltsstoffe | 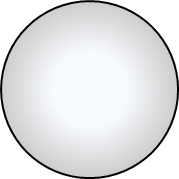 | 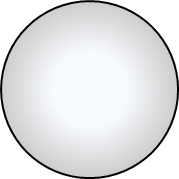 | 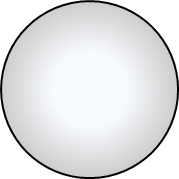 | 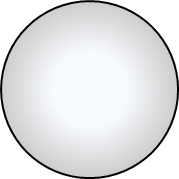 | 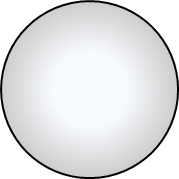 |
