# Supplementary figures and images for: Physicians’ beliefs about placebo and nocebo effects in antidepressants – an online survey among German practitioners
Source: PLoS One. 2017 May 31;12(5):e0178719. doi: 10.1371/journal.pone.0178719 (PMC5451122; doi:10.1371/journal.pone.0178719)

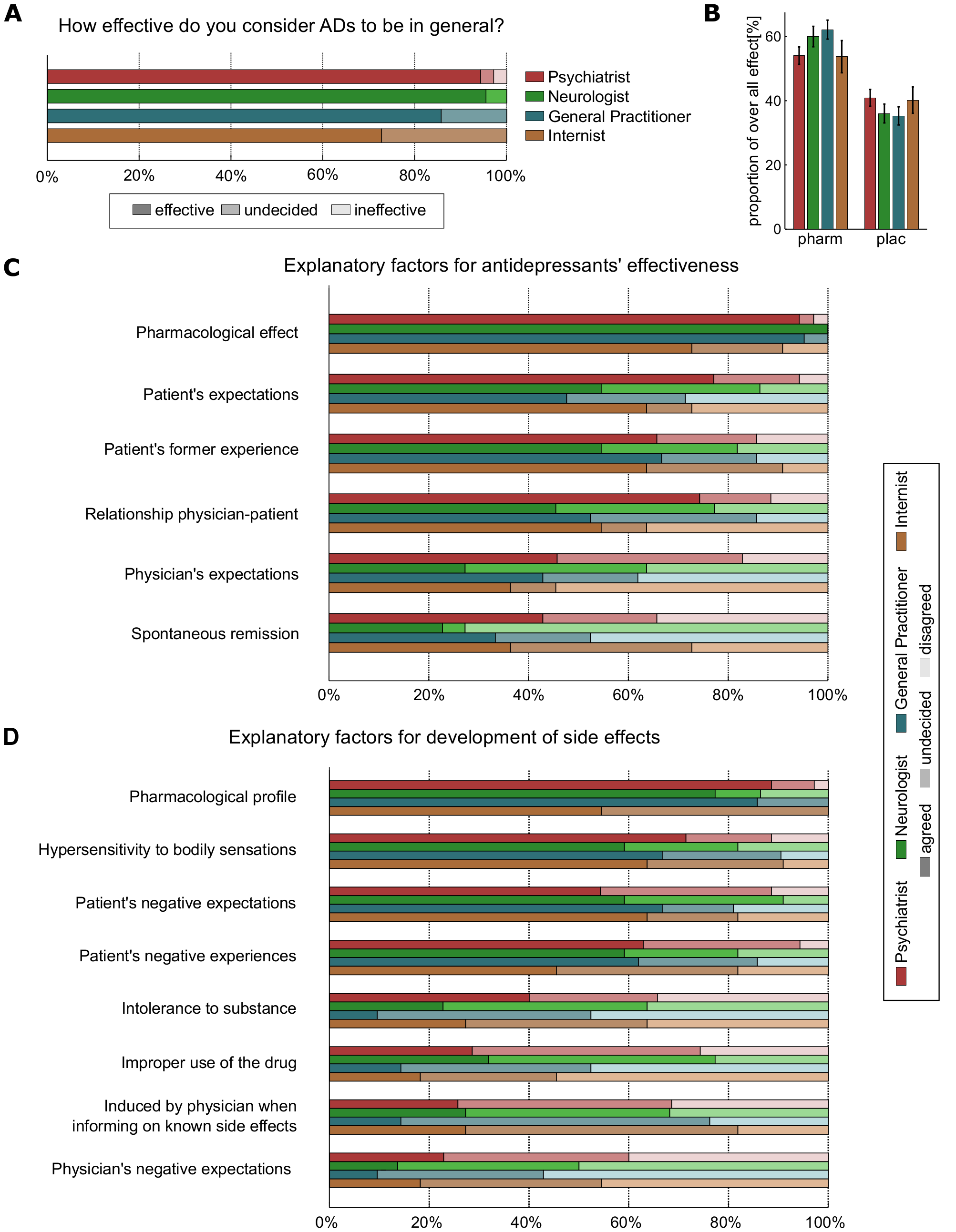

Supplement: S1 Fig — Color coding was chosen to differentiate between specialties of participants, where red = psychiatry (n = 35), green = neurology (n = 22), blue = general medicine (n = 21) and brown = internists (n = 11), whereas these colors were shaded to code agreement (darker) vs. disagreement (brighter), just as introduced in Figs 1 and 2 of the result section. (A) Ratings on general effectiveness of antidepressants (AD) (agreed, undecided, disagreed) (B) Estimated proportion of pharmacological (pharm) vs. placebo (plac) responses in ADs' overall effectiveness ([%], M ± SEM). (C) Endorsement of six different explanatory factors for ADs' effectiveness. (D) Rated relevance of eight explanatory factors for the development of side effects in AD. (TIF) [file pone.0178719.s003.tif]
